# Insights of *Phaseolus vulgaris*’ response to infection by *Uromyces appendiculatus* using an RNA-seq approach

**DOI:** 10.3389/fpls.2025.1557954

**Published:** 2025-06-02

**Authors:** Penny Makhumbila, Molemi Rauwane, Hangwani Muedi, Sandiswa Figlan

**Affiliations:** ^1^ Department of Agriculture and Animal Health, School of Agriculture and Life Sciences, College of Agriculture and Environmental Sciences, University of South Africa, Roodeport, South Africa; ^2^ Department of Botany, Nelson Mandela University, Port Elizabeth, South Africa; ^3^ Research Support Services, North-West Provincial Department of Agriculture and Rural Development, Potchefstroom, South Africa

**Keywords:** common bean rust, transcriptomics, gene expression, differentially expressed genes, biomarkers

## Abstract

Rust, caused by the obligate biotrophic fungal pathogen *Uromyces appendiculatus* (Pers.:Pers.) Unger, is one of the most destructive diseases of common bean (*Phaseolus vulgaris* L.) causing yield losses in production areas worldwide. This study explores the use of RNA sequencing (RNA-seq) as a breeding prediction tool through the assessment of gene expression patterns of common bean susceptible (Golden Gate Wax) and resistant (Teebus-RR-1) varieties to *U. appendiculatus* virulent strain race 31-1 at two time points (14- and 21- days post infection; dpi). *U. appendiculatus* induced stress-responsive genes such as heat shock proteins (HSPs: *HSP17.6II* and *ATHSP22.0*), receptor-like kinases (RLKs: *CRK29*), cytochrome monooxygenases (*CYP76C4*, *CYP82C4* and *CYP94B1*) and terpene synthases (*TPS21* and *TPS14*) at 14-dpi, while *BIA* and *WRKY70* transcription factors, among others were regulated differentially in both varieties at 21-dpi. Genes *RPS2*, *CAR1* and *DM2H* were among the identified potential biomarkers associated with *U. appendiculatus* resistance. Enrichment of signaling receptor activity, response to stress, sesquiterpenoid and triterpenoid biosynthesis, and plant hormone signal transduction were unique to Teebus-RR-1. Overall, the findings of this study indicate varying gene expression patterns between varieties in response to *U. appendiculatus*. Insights provided by the RNA-Seq approach used in this study on mechanisms of *P. vulgaris’* response to *U. appendiculatus* can be used for biomarker discovery and possible development of resistant varieties.

## 1 Introduction

Common bean (*Phaseolus vulgaris* L.) is a principal legume crop that is important in many households of developing countries as a primary source of protein, vitamins and minerals (Carbas et al., 2020). The crop contributes economically and socially to populations in Latin America, Eastern and Southern Africa (Vasconcelos et al., 2020). Several diversified products such as gluten free flour used for products including biscuits with low antinutrient content, spaghetti with a higher concentration of phenolic compounds and bread with increased nutritional and mineral composition have been developed from the crop (Carbas et al., 2020). Despite the wide cultivation of *P. vulgaris* worldwide, it is constrained by extreme abiotic and biotic stressors that are often exacerbated by the ever-changing climatic factors. The frequent excessive rainfall and humid conditions that tend to occur during crucial stages of plant development increase the occurrence, prevalence and severity of pests and pathogens (Brescia et al., 2023).

Rust caused by the fungal pathogen *Uromyces appendiculatus* (Pers.:Pers) Unger. poses a major threat to the productivity of legumes worldwide. The constant occurrence of the disease in temperate, tropical, and subtropical regions of the Americas and Africa has resulted in major crop losses (Monteagudo et al., 2006). The pathogen’s main host is *P. vulgaris* and other *Phaseolus* spp. such as runner bean (*Phaseolus coccineus*), lima bean (*P. lunatus*) and cowpea (*Vigna unguiculata*). Upon infection, the pathogen penetrates the stomata, then the mesophyll cells and the haustorium forms in the cell cavity (RoyChowdhury et al., 2022). Post infection, the uredinia (brown rust-like spots) appear and enlarge over time on the primary leaves, which may further spread to the secondary leaves, stems, and pods (Leitão et al., 2023). When the conditions are highly favorable for the pathogen, leaf yellowing, senescence and premature leaf abscission are prevalent, consequently impacting the yield of the crop (Wafula et al., 2023).

Cultural practices such as deep cultivation and long-term rotation with non-host crops such as maize (*Zea mays*) and sorghum (*Sorghum bicolor*) have been used to curb infestation by pathogens in legume production systems (Montejo Domínguez et al., 2022). *U. appendiculatus* can be controlled using fungicides which have been greatly criticised for their negative impact on the environment, human and animal health (Shtaya et al., 2021). However, the pathogen is rapidly evolving and thus developing unique virulence, leading to poor success in its control (Abo-Elyousr et al., 2021). Deployment of varieties with known polygenic pool of *U. appendiculatus* resistant genes identified through high throughput technologies is critical to ensure stable yields across diverse pathogen races, consequently reducing intensive chemical use. There have been numerous efforts in breeding varieties for rust resistance, including the identification of genes imparting resistance to the pathogen (Wafula et al., 2023). To date, 14 dominant genes have been mapped to confer resistance to rust in the genome of *P. vulgaris* (Schmutz et al., 2014), namely *Ur-3* (*Pv11*), *Ur-5* (*Pv04*), *Ur-7* (*Pv11*), *Ur-11* (*Pv11*) and *Ur-14* (*Pv04*) that belong to the Mesoamerican gene pool. Genes *Ur-4* (*Pv06*), *Ur-6* (*Pv11*), *Ur-9* (*Pv01*), *Ur-12* (*Pv07*) and *Ur-13* (*Pv08*) belonging to the Andean gene pool were also characterised thus contributing greatly to breeding for *U. appendiculatus* tolerance (**Supplementary Table S1**; Arunga et al., 2012; Leitão et al., 2023; Devi et al., 2024). While single-gene resistance is often easier to characterize, it rarely provides conclusive or lasting protection against diverse pathogen races and frequently fails to withstand evolutionary pressures (Valentini et al., 2017). Furthermore, studies reporting on resistant genes of *P. vulgaris* in response to *U. appendiculatus* don’t provide a broad overview of all genes responsible for plant-pathogen interaction. Although previous efforts have resulted in improved varieties, it would be beneficial for breeding programmes to target multiple genes that confer broad spectrum resistance to the pathogen. Additionally, knowledge of the molecular mechanisms underlying resistance to *U. appendiculatus* is still limited. This study aimed to evaluate *P. vulgaris’* response to a virulent strain of *U. appendiculatus* (race 31-1) at different time intervals. The identification of differentially expressed genes (DEGs) together with potential biomarkers provided by this study can be used as a basis to understand the underlying molecular processes involved in *U. appendiculatus* stress response, thus accelerating breeding efforts geared at improving *U. appendiculatus* resistance in common bean.

## 2 Methods

### 2.1 Plant materials, experimental design and fungal inoculation

Two common bean varieties susceptible (Golden Gate Wax) and resistant (Teebus-RR-1) to *U. appendiculatus* were obtained from the Agricultural Research Council – Grain Crops Institute (ARC-GCI), Potchefstroom, South Africa. Golden Gate Wax previously characterised to have rust resistant gene *Ur-6* (conferring resistance to race 47-1) and Teebus-RR-1 previously characterised to have rust resistant gene *Ur-3* (conferring resistance to races 31-1, 38-1 and 47-1) and *Ur-6* (Park et al., 2008; Madubanya et al., 2009). Golden Gate Wax and Teebus-RR-1 have been widely used as parental lines in breeding programmes for their wide range of phenotypic characteristics (Valentini et al., 2017). Seeds of both varieties were surface sterilised using 50% bleach solution (Gilbert et al., 2023), rinsed with double distilled water, pre-germinated in Petri dishes (Lasec, South Africa) and then grown in pots with two seeds per pot (9 cm diameter) using 30 dm^3^ sterile seedling mix constituting compost and topsoil, topped with vermiculite to cover seeds. Seedlings were treated twice with multi-feed water soluble fertiliser (19:8:16, NPK) with other active macro- and micro- nutrients (Efekto, South Africa).

Prior to conducting this study, several varieties and rust races were evaluated. Race 31-1, a widely distributed strain of *U. appendiculatus* in common bean production areas of South Africa was found to be virulent to several susceptible varieties upon evaluation in the early vegetative stage (14-dpi; **Supplementary Figure 1**) and therefore was selected for this study. Previously isolated, purified and characterised *U. appendiculatus* race 31-1 urediniospores stored in a -80°C ultra-freezer (Arunga et al., 2012) were obtained from the ARC-GCI. The urediniospores were re-hydrated in a warm glass beaker bath with vermiculite while the pathogen cryotubes were opened and sealed with sterile cling wrap to allow the hydration of spores. The re-hydration process of the spores was done at room temperature (±18°C) for 12 h. The spore suspension was then prepared in an Erlenmeyer flask with 60 mg of spores, 50 ml distilled water mixed with Tween 20 (P1379, Sigma-Aldrich, Merck, United States; 5 drops per 1 L; Abo-Elyousr et al., 2021).The solution was vigorously shaken, and concentrations adjusted to 2.5 x 10^4^ using a haemocytometer. Upon the full development of the first trifoliate leaves, the plants were inoculated by spraying the spore suspension on the underside of the leaf at a pressure of 30 kPA, and approximately +0.5 ml of the suspension was used for each plant to cover the full leaves. After drying, the plants were placed in a humidity chamber with light and 95-100% relative humidity created by distilled water at temperatures of ±18–20°C for 48 h. The control experiment was “mock” inoculated with distilled water and subjected to the same conditions as *U. appendiculatus* inoculated. After incubation, all plants were moved to benches in the greenhouse with 28/14°C day/night temperatures and 75% relative humidity. The plants were then transferred to 50 L grow bags with sterile oxidic soii, two days after removal from the humidity chamber. To avoid sampling on the same plant at different experimental time points, which may cause gene expression variations, three greenhouse replications, with 5 plants per 50 L bag were planted in a randomised complete block design (RCBD) for each time point and variety (Hartung et al., 2019). Furthermore, the plants were placed in different compartments to separate the treated and control experiments prior to evaluation (Villegas-Fernández et al., 2023).

### 2.2 Phenotypic evaluation, RNA extraction and sequencing

Phenotypic evaluations of the varieties were done at 14- and 21-days post-infection (dpi). Evaluating later time points (14 and 21-dpi) helps uncover sustained plant resistance mechanisms and stage-dependent susceptibility. For obligate biotrophs such as *U. appendiculatus*, these stages are critical, as they align with spore production and possible host resource manipulation (Thibivilliers et al., 2009; Voegele and Mendgen, 2011). Varieties Golden Gate Wax and Teebus-RR-1 were phenotypically evaluated throughout the duration of the experiment, with specific interest in two time points 14- (early vegetative stage) and 21-dpi (pre-flowering stage; Makhumbila et al., 2023). The rust severity on the plants was observed based on the pustule size and scored at both time points utilising the uredenia score criteria (Valentini et al., 2017). At both time points of the experiment, 3 replicates of leaf samples were harvested randomly from all treatments and snap-frozen in liquid nitrogen prior to total RNA extraction. Total RNA was extracted using a ZymoBIOMICS (Zymo Research, USA) kit and RNA cleanup was done using RNeasy (Qiagen, USA) kit for 24 samples. The quality and concentration of the RNA samples were evaluated using a Qubit^®^ fluorometer (ThemoFisher Scientific, USA). The library preparation and sequencing were conducted at the Agricultural Research Council – Biotechnology Platform (South Africa) using an MGI DNBSEQ-G400 (MGI Tech, China) instrument.

### 2.3 Data analysis

The raw data obtained was assessed in FastQC v0.11.5 and trimmed to remove adaptors and ambiguous sequences using TrimGalore v0.6.5 (Krueger, 2015).The Plant Genomics Resource (JGI) *Phaseolus vulgaris* v2.1 (https://phytozome-next.jgi.doe.gov/info/Pvulgaris_v2_1) was used as a reference genome to align and assemble the trimmed data using HISAT2 v2.0.6 (Kim et al., 2019). Samtools v1.9 (Danecek et al., 2021) was used to view and sort the data before the assembly of RNA-Seq alignments to potential transcripts in StringTie v2.2.1 (Shumate et al., 2022). The bioinformatics workflow and parameters used in this study have been described (Van den Berge et al., 2019). The gene expression matrix obtained from StringTie were analysed in DESeq2 v1.42.1 (Love et al., 2014) to test for differentially expressed genes (DEGs) employing the negative binomial distribution with P-adjust ≤ 0.05 and |log_2_FC| ≥1. The normalised gene counts from DESeq2 were further analysed using SIMCA v18 (Umetrics, Sweden) for sample patterns and biomarker discovery using an orthogonal partial least squares (OPLS) model. Functional enrichment/biological category analysis was conducted in g:Profiler with P-adjust ≤ 0.05 (Raudvere et al., 2019). The raw data was submitted into NCBI Sequence Read Archive (SRA) with bio project ID: PRJNA1061833.

### 2.4 Validation of DEGs by qRT-PCR analysis

Quantitative real-time polymerase chain reaction (qRT-PCR) analysis was performed from leaf samples of *P. vulgaris* harvested at 14 and 21-dpi. Only seven (7) genes were common across treatments and Five (5) genes were selected from the common genes for quantitative real-time (qRT) PCR analysis. Additionally, two (2) biomarkers were selected for downstream qRT-PCR. A common practice in gene expression research is to focus on shared genes across treatments, as this reduces variability and strengthens the study’s reliability (Li et al., 2022). Primers for the selected DEGs and potential biomarker genes were designed using NCBI’s primer blast (https://www.ncbi.nlm.nih.gov/tools/primer-blast/; **Table 1**). Primers included amplicon length less than 200 bp, melting temperatures of 50 – 60 °C and GC content of 40 - 60%. The reverse transcription (RT) of RNA was conducted using Takara’s PrimeScript 1st strand cDNA synthesis kit in accordance with the manufacture’s protocol (Takara Bio, Europe, France). The concentrations of complementary DNA (cDNA) were quantified using the Qubit ^®^ fluorometer, and equal concentrations of 50 ng/μL were prepared for qRT-PCR. Complementary DNA samples were sent to Inqaba Biotec (Inqaba Biotechnical Industries, South Africa) for qRT-PCR analysis using the CFX 96 Real-Time PCR System (Bio-Rad, Hercules, CA). The internal reference gene, common bean *Actin*, was utilized to normalize the Ct values of every reaction (Montero-Tavera et al., 2017). Relative expression was calculated using the ^ΔΔ^Ct method (Harshitha and Arunraj, 2021).

**Table 1:** Primers of targeted DEGs, biomarkers and house keeping genes used for qRT-PCR analysis.

Group	Unigene	Gene	F	R	Amplicon size (bp)
DEGs	Phvul.007G203400	GolS2	GTGCTGCGGTGAGGGTTAAT	CCCACTGTACCTCCAAGGCT	114
	Phvul.008G016500	BAG5	CCATGAGGGTGGAGTTGGCA	CTTTCTGCACTCCCTGAGGC	164
	Phvul.009G078300	HSFA2	GCGTCCAAACCCTAGTGGAC	AGCAACCACCTTCCCTGCTG	192
	Phvul.002G155300	HSFB2B	CGTCGCAAGAACCAGATGGC	GTGCAAAGACTGCTCGCACA	174
	Phvul.001G154700	HSFA6B	ACCACTGAAACCCCACTTTGT	ACCGACAAGGTTAACCACCA	196
Biomarkers	Phvul.006G055950	ABO4	AGGCTGGAGAACTAAAGGCTTG	GGAATATAAAATTTCAGCCGCAAC	189
	Phvul.010G054700	DM2H	TTAAGGTGCCTTGAACGGCT	GGTTTGCAAAGGGAGAACGG	141

## 3 Results

### 3.1 Phenotypic variations between varieties infected with *U. appendiculatus*

**Figure 1:**
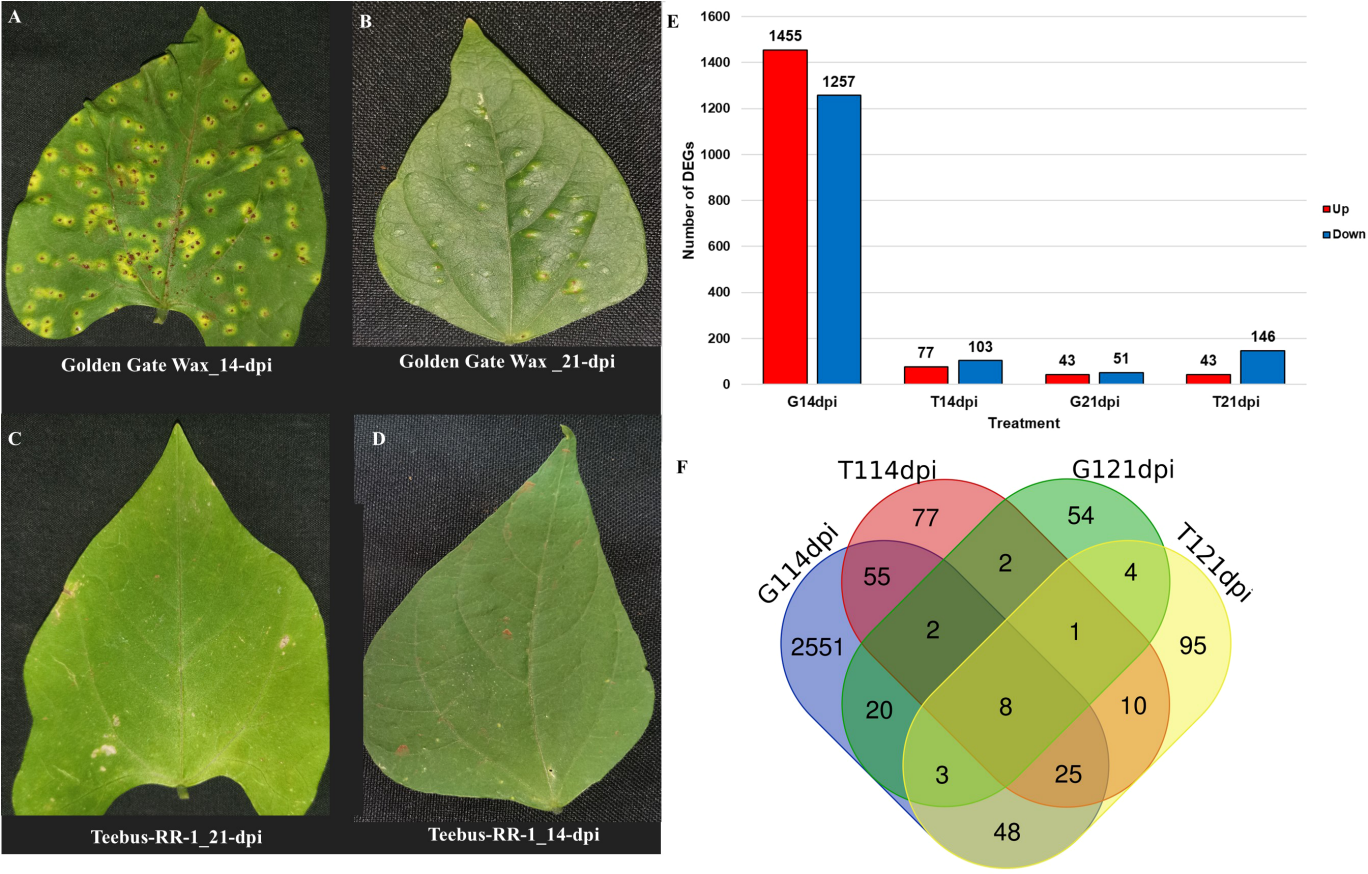
Overview of phenotypic evaluation and DEGs of common bean varieties infected with race 31-1 of *U. appendiculatus*. Golden Gate Wax leaf at 14-dpi **(A)**; Golden Gate Wax leaf at 21-dpi **(B)**; Teebus-RR-1 leaf at 14-dpi **(C)**; Teebus-RR-1 leaf at 21-dpi **(D)**; Number of up- and down-regulated DEGs between Golden Gate Wax and Teebus-RR-1 at both time points of evaluation **(E)**; Venn diagram analysis of DEGs between Golden Gate Wax and Teebus-RR-1 at both time points of evaluation **(F)**. Denotations on figure are G: Golden Gate Wax, T: Teebus-RR-1, 14dpi: 14 days post infection, 21dpi: 21 days post infection and digit after variety symbol indicates race 31-1).

Golden Gate Wax and Teebus-RR-1 varieties with varying response to *U. appendiculatus* were evaluated for pathogen severity in glasshouse compartments at 14- and 21-dpi respectively. Golden Gate Wax scores showed prominent symptoms at 14- and 21-dpi (**Figures 1A, B**), while Teebus-RR-1 exhibited no symptoms post-infection with *U. appendiculatus* at both time points (**Figures 1C, D**). In addition, at 21-dpi, the pathogen had progressed greatly to infecting the secondary leaves (secondary re-infection) of Golden Gate Wax and primary leaves had defoliated (**Figure 1B**). The phenotypic observations made in this study indicated resistance in Teebus-RR-1 while Golden Gate Wax showed susceptibility to the pathogen at both time points.

### 3.2 RNA-seq statistics

Over 90 million raw sequence reads were obtained from data of both varieties at both time points (**Supplementary Table S2**). Ninety percent of the reads mapped to the *P. vulgaris* v2.1 reference genome. The multivariate statistical analysis tool, viz OPLS model was also applied to the data to screen variations between treatments and gene expression importance (**Supplementary Figure S2**).

### 3.3 Transcriptome mapping of *P. vulgaris*’ response to *U. appendiculatus*

**Figure 2:**
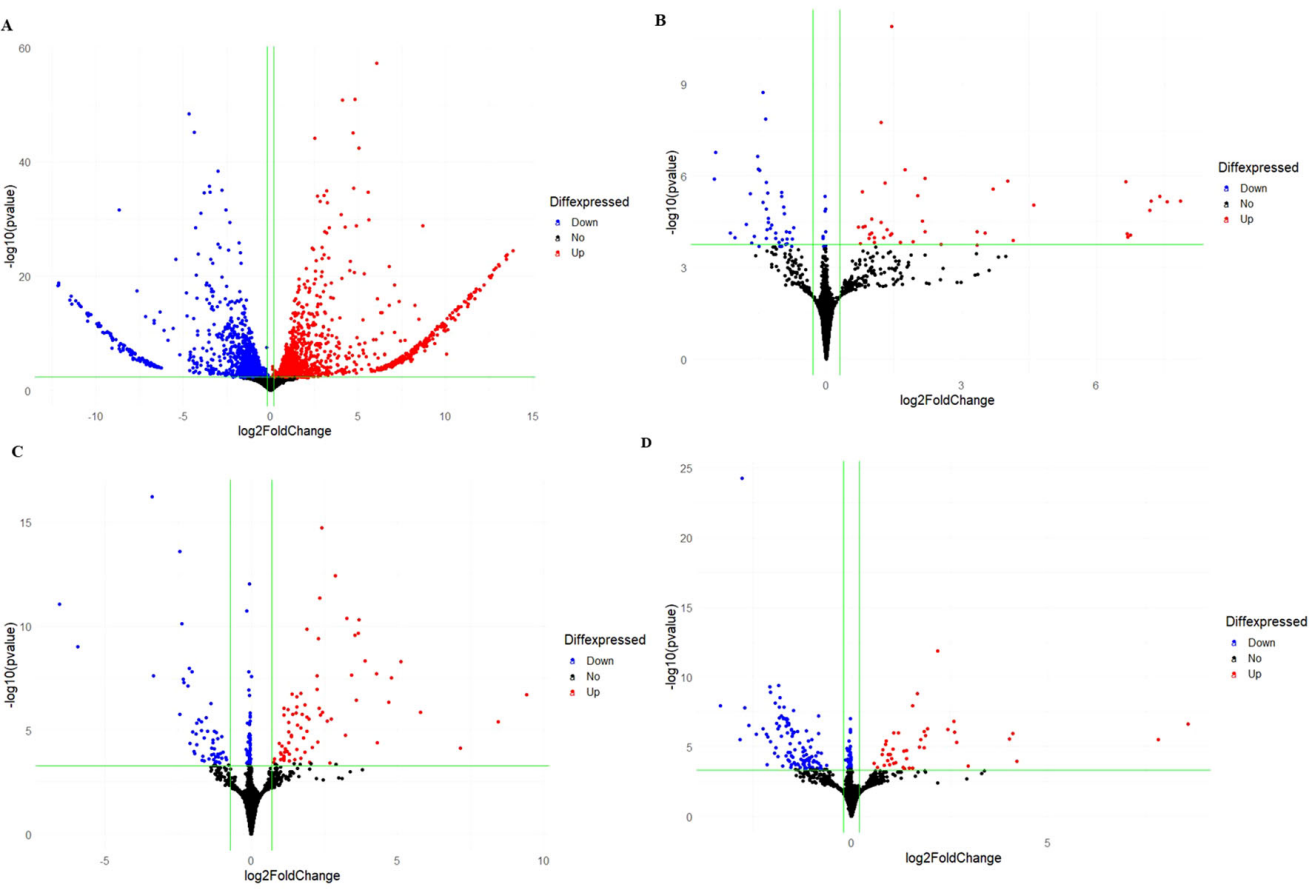
Volcano plots comparing the DEGs expressed in both varieties at 14- and 21-dpi. Gene regulation in Golden Gate Wax at 14-dpi **(A)**; Gene regulation in Golden Gate Wax at 21-dpi **(B)**, Gene regulation in Teebus-RR-1 at 14-dpi **(C)**; Gene regulation in Teebus-RR-1 at 21-dpi **(D)**. Blue colour represents genes that were significantly down-regulated, red represents genes that were significantly up-regulated and black indicates genes that were not significantly expressed.

A total of 3175 DEGs were observed between the two varieties for all the treatments, with Golden Gate Wax expressing 88% (2806) of the total DEGs while Teebus-RR-1 revealed only 369 DEGs (**Figures 1E, F**). At 14-dpi, 1455 and 1257 DEGs were respectively up- and down-regulated in Golden Gate Wax (**Figures 1E**, **2A**), while 77 and 103 DEGs were detected in Teebus-RR-1 under the same conditions (**Figures 1E**, **2C**). At 21-dpi, Golden Gate Wax had 94 DEGs, 43 of which were up-regulated (**Figures 1E**, **2B**) and 51 were down-regulated, while Teebus-RR-1 had 189 DEGs, of which 43 were up-regulated and 146 down-regulated (**Figures 1E**, **2E**). In addition, 8 genes were found to be commonly expressed between the treatments for both varieties (**Figure 1F**) and 7 of these genes were used to design primers for qRT-PCR analysis (**Table 1**, **Supplementary Table S4**). Generally, there was a high expression of genes in Golden Gate Wax compared to Teebus-RR-1 (**Supplementary Figure S3**).

### 3.4 Gene expression variations of *P. vulgaris’* response to *U. appendiculatus* infection

DEGs were classed into gene families. At 14-dpi, several genes and transcription factors were involved in regulating stress caused by *U. appendiculatus’* infection. Response mechanisms of common bean varieties included the expression of gene families such as heat shock proteins (HSPs), receptor-like kinases (RLKs), enzyme cytochrome monooxygenases and terpene synthases, among others (**Supplementary Table S3**). For example, infection with *U. appendiculatus* race 31-1 revealed the expression of heat shock proteins (*HSP17.6II*: *Phvul.001G039700*, *Phvul.001G039800*, *ATHSP22.0*: *Phvul.004G129400*, *HSP17.6C*: *Phvul.008G228000*, *HSP15.7*: *Phvul.008G237000*, *HSP17.8*: *Phvul.009G152500*), cystine-rich RLK (*CRK29*: *Phvul.007G049500*, *Phvul.007G049600*), cytochrome P450 family genes (*CYP76C4*: *Phvul.007G106300*, *CYP82C4*: *Phvul.004G022000*, *CYP94B1*: *Phvul.001G158100*), and terpene synthase family (*TPS14*: *Phvul.002G219300*, *TPS21*: *Phvul.011G143100*) in both varieties of *P. vulgaris* at 14-dpi. At 21-dpi, rust infection resulted in the expression of a peptide that enhances stress-induced cell death (*BIA*: *Phvul.010G019701*), which was down-regulated in Golden Gate Wax, while up-regulated in Teebus-RR-1. Furthermore, *WRKY70* (*Phvul.008G081800*) transcription factor, a DNA binding protein was down-regulated in Teebus-RR-1 while up-regulated in Golden Gate Wax (**Supplementary Table S3**). Other common DEGs expressed in both varieties at 14- and 21-dpi belonged to the galactinol synthase (*GolS1*: *Phvul.001G215300*) and BCL-2-associated athanogene (*BAG5*: *Phvul.008G016600*) families among others (**Supplementary Table S4**). Interestingly, multiprotein bridging factor 1C gene *MBF1C* (*Phvul.004G162100*) and isomerase gene *ROF2* (*Phvul.003G064500*) were up-regulated in Teebus-RR-1 and down-regulated in Golden Gate Wax at 14-dpi, while they were not expressed at 21-dpi (**Supplementary Table S3**). The expression of the latter genes has been reported to enhance resistance to both biotic and abiotic stresses, suggesting their role in Teebus-RR-1 in response to rust infection. The expression patterns of the targeted genes selected were consistent with the qRT-PCR analysis (**Supplementary Figure S4**).

### 3.5 Potential biomarkers associated with *P. vulgaris’* resistance against *U. appendiculatus*

**Figure 3:**
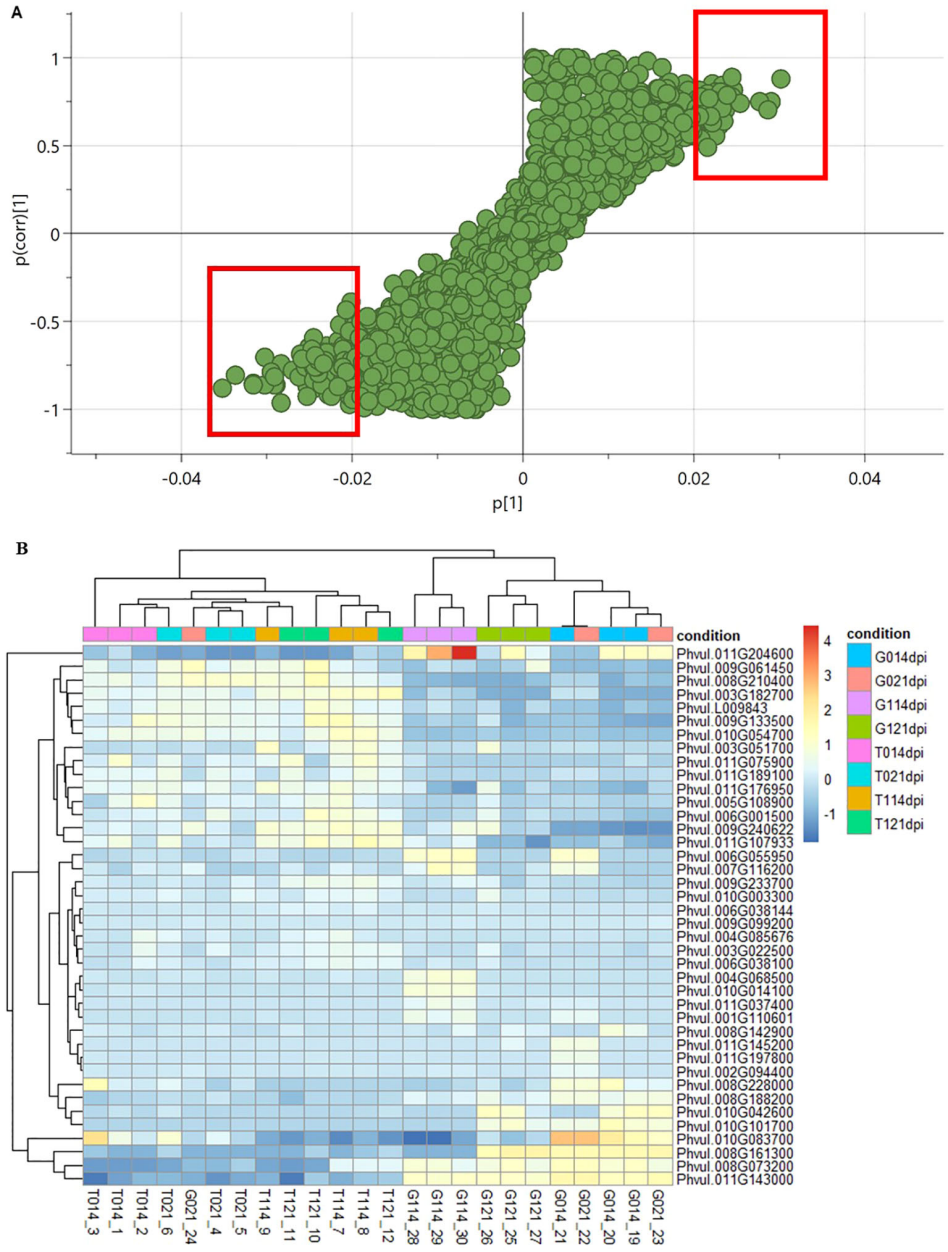
Plots indicating potential biomarkers associated with resistance to *U. appendiculatus* race 31-1. S plot derived from the OPLS model indicating potential biomarker genes with upper and lower biomarkers highlighted in red **(A)**. Heatmap of identified biomarkers represented as unigenes expressed in both varieties for all the treatments **(B)**. The upper colours on the heatmap denote the sample treatments, race 31-1 is denoted as 1, and control denoted as 0. Golden Gate Wax is denoted as (G) and Teebus-RR-1 denoted as (T) at both time points of evaluation (14- and 21-dpi).

To identify potential biomarkers associated with *U. appendiculatus* resistance in *P. vulgaris*, normalised gene counts were used to draw an S-plot derived from the OPLS model (**Figure 3A**). Variables that were on the extreme lower left and upper right quadrants were identified as putative biomarkers (**Supplementary Table S5**). In addition, the data was subjected to Variable Importance Projection (VIP) and only biomarkers with VIP ≥1 annotated in the *P. vulgaris* genome were considered, for the top right- and lower left quadrant (Li et al., 2020; **Supplementary Table S5**). Furthermore, to explore potential biomarkers, cluster heatmaps were computed to indicate the gene expression patterns of *P. vulgaris* unigenes across treatments (**Figure 3B**). There were variations in gene expression levels between the two varieties evaluated at both time points post infection with *U. appendiculatus* race 31-1. Gene expression levels of potential biomarkers in Golden Gate Wax were moderately higher compared to Teebus-RR-1 (**Figure 3B**). Interestingly, biomarker genes expressed in Golden Gate Wax had distant clustering (eg, *Phvul.011G143000* – *Phvul.010G083700*) compared to biomarker genes expressed in Teebus-RR-1.

The potential biomarker genes were further classed into families to identify expression patterns or family groups expressed as a stress response strategy against *U. appendiculatus*. Like patterns observed in DEGs, several genes and transcription factors showed involvement in the regulation of the rust pathogen. Potential biomarkers from the top right quadrant induced included genes from families such as nucleotide-binding (NB-ARC) domain, leucine-rich repeats (LRR-containing) domain and cytochrome monooxygenases (CYP450 family), among others (**Supplementary Table S5**). Interestingly, NB-ARC domain-containing disease resistance proteins (*RPS2*: *Phvul.006G038100*, *CAR1*: *Phvul.009G233700*) were variably down-regulated in both varieties at both time points. In addition, an *LRRAC1* (*Phvul.011G197800*) disease resistance protein was repressed in Teebus-RR-1 at both time points, while greatly up-regulated in a Golden Gate Wax with a log2FC > 3. Strikingly, another *TIR-NBS-LRR* class protein *DM2H* (*Phvul.010G054700*) was expressed in both varieties at varying levels. Furthermore, notable genes expressed included DNA polymerase epsilon catalytic subunit (*ABO4: Phvul.006G055950*) and disease resistance protein (TIR-NBS-LRR class) family (*DM2H*: *Phvul.010G054700*). Genes from the bottom left quadrant identified as potential biomarkers included the thiolase protein family (*AACT1*: *Phvul.008G161*300), ribonuclease P /Rpp14 family protein (*EMB1687*: *Phvul.008G188200*), *HSP20*-like chaperones superfamily protein (*HSP17.6C*: *Phvul.008G228000*) and zinc finger CCCH domain protein (MADA4: Phvul.010G101700; **Supplementary Table S5**). Furthermore, the expression patterns of identified biomarker genes *ABO4* (*Phvul.006G055950*) and *DM2H* (*Phvul.010G054700*) were consistent with results obtained from qRT-PCR analysis (**Supplementary Figure S4**).

### 3.6 Functional annotation of *U. appendiculatus* responsive genes

**Figure 4:**
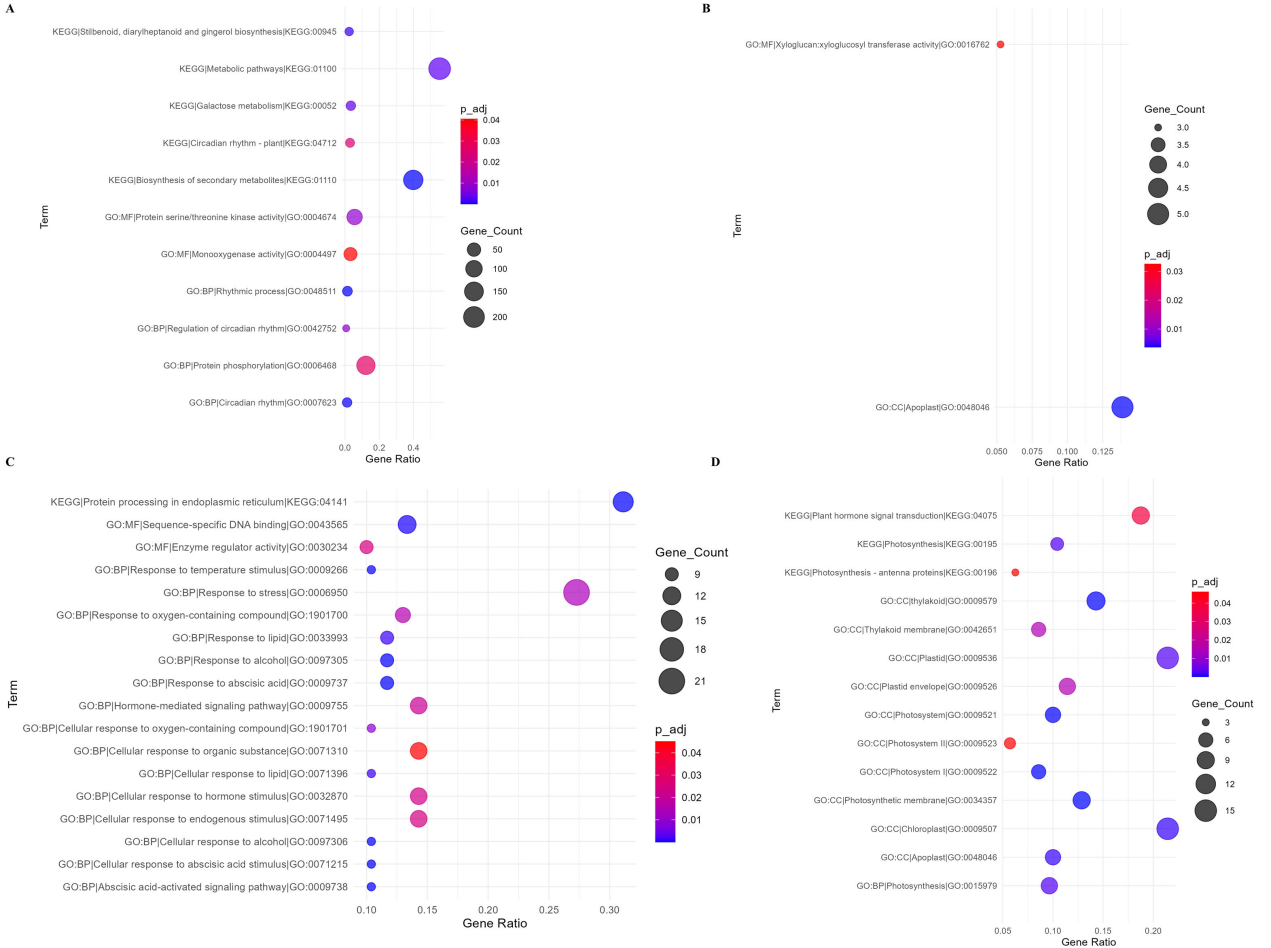
Gene ontology terms enriched in *P. vulgaris* in response to *U. appendiculatus*. Enriched terms in Golden Gate Wax at 14-dpi **(A)**; Enriched terms in Golden Gate Wax at 21-dpi **(B)**; Enriched terms in Teebus-RR-1 at 14-dpi **(C)** and Enriched terms in Teebus-RR-1 at 21-dpi **(D)**. Circle size on plot represents gene count impact on enriched term and circle colour indicating significance of enriched term (P-adjust ≤ 0.05).

To identify the functional categories of DEGs, Gene Ontology (GO) and Kyoto Encyclopedia of Genes and Genome (KEGG) enrichment analysis was conducted in g:Profiler (https://biit.cs.ut.ee/gprofiler/gost; Reimand et al., 2019). Enriched pathways with more than 2 gene counts were examined and dot plots were computed in R (ggplot2; Gómez-Rubio, 2017) with *P-adj* ≤ 0.05. The dot plots demonstrated the influence of genes on Biological Functions (BF), Cellular Components (CC) and Molecular Functions (MF) of the DEGs as well as the KEGG pathways (**Figure 4**).

In Golden Gate Wax at 14-dpi, metabolic pathways (KEGG:01100) and biosynthesis of secondary metabolites (KEGG: 01110) were statistically enriched (**Figure 4A**). Additionally, MF protein serine/threonine kinase activity (GO: 004674) and BP, protein phosphorylation (GO:0006468) were enriched by over 50 DEGs among other pathways (**Figure 4A**). Interestingly, at 21-dpi, MF, xyloglucan:xyloglucosyl transferase activity (GO:0016762) and CC, apoplast (GO:0048046) were the only pathways enriched by Golden Gate Wax (**Figure 4B**). On the other hand, Teebus-RR-1 at 14-dpi enriched protein processing in the endoplasmic reticulum (KEGG: 04141), MF, sequence-specific DNA binding (GO:0043565), BP, response to stress (GO:0006950), hormone mediated signaling (GO: 0071310), cellular response to organic substance (GO: 0071310), cellular response to hormone stimulus and cellular response to endogenous stimulus (GO: 0071495) pathways among others (**Figure 4C**). Intriguingly, plant hormone signaling transduction (KEGG: 04075), CC, thylakoid (GO: 0009579), photosynthetic membrane (GO: 0034357) and chloroplast (GO: 0009507) among other pathways were statistically enriched by Teebus-RR-1 at 21-dpi (**Figure 4D**).

## 4 Discussion

The prevalence of *U. appendiculatus* in production areas of *P. vulgaris* causes significant damage at critical growth stages of the crop, ultimately resulting in yield losses (Leitão et al., 2023). Therefore, the development of varieties with resistance to *U. appendiculatus* should be key in breeding programmes (Bosamia et al., 2020). The availability of the *P. vulgaris* reference genome provides a foundation for discovering transcription factors that might be responsible for pathogen resistance. Several studies have used the RNA sequencing (RNA-seq) approach to understand the interaction of legumes with plant pathogens (Chen et al., 2020; Jiang et al., 2021). However, limited reports have utilised transcriptome analysis to assess gene expression patterns in *P. vulgaris* infected by the *U. appendiculatus* virulent strain race 31-1 at critical plant growth stages (early vegetative stage and pre-flowering). Hence the aim of this study was to evaluate the response of common bean varieties to the virulent strain of *U. appendiculatus* race 31-1 at two critical time-points.

### 4.1 Gene regulation variations in *P. vulgaris* varieties in response to *U. appendiculatus*

Several gene families associated with defense have been characterised in plants, and the expression of HSPs/chaperones has been found to be associated with plant-pathogen interaction, among other functions (Garofalo et al., 2009). In occurrences where plants are under pathogen attack, HSPs can prevent protein aggregation, translocation across cell membranes and degradation, thus consequently preventing the negative impacts of pathogen stress (Katam et al., 2020). HSP20 gene family has been characterised as essential proteins that are important for general plant growth and development. In addition, the plant’s regulation of HSPs has also been associated with several cell functions that stabilise and refold certain proteins as a plant immunity strategy by plants (Singh et al., 2022). In this study, the *HSP17.6II* was down-regulated in Golden Gate Wax, while up-regulated in Teebus-RR-1. Similar results were observed in tomato (*Solanum lycopersicum*) plants infected with early blight (*Alternaria solani*), where *HSP17.6II* was down-regulated, signifying a specialised response strategy in varieties (Upadhyay et al., 2014). The up-regulation of specific HSPs/chaperones such as *HSP17.6C* has also been found to be important for heat acclimation in *Arabidopsis* (Yamaguchi and Ito, 2021). In the current study, *HSP17.6C* was up-regulated in both varieties at 14-dpi. However, it was up-regulated by two folds in Teebus-RR-1 in comparison to Golden Gate Wax. This may indicate that, although varieties may regulate genes similarly, abundance in gene expression levels may signify a specific effect in response to stress (Plett and Martin, 2018). Similar expression patterns of *HSP17.8* have previously been observed (Grigorova et al., 2011). The significant up-regulation of *HSP17.8* has been previously associated with an increase in plant immunity, protein stability and inhibition of programmed cell death (Bolhassani and Agi, 2019). HSPs such as, *HSP17.6II*, *HSP17.6C*, and *HSP17.8* among others have been found to contribute to plant-pathogen interaction (de Souza Resende et al., 2022; Zhang et al., 2024). However, certain fungal pathogens exploit them as effectors to manipulate plant immunity (Fabro, 2022). For example, in soybean (*Glycine max*) infected with *Phytophthora sojae*, overexpression of *HSP17.6C* was found to be correlated with susceptibility, thus suggesting pathogen-induced co-option of host HSPs (Wang et al., 2011). In this study, Teebus-RR-1 showed an overexpression of *HSP17.6C* and we hypothesize that this may represent a decoy strategy, inhibiting the pathogen’s ability to co-opt this gene for virulence. More studies are required to future dissect individual roles of HSPs in plant-pathogen interactions.

The molecular activities of *RLK* gene family have been found to be activators of LRR and intracellular kinase domains that regulate the development, growth and stress response in plants. The up-regulation of RLK genes such as CRK29 upon pathogen perception has been found to be associated with resistance. This was consistent with the results of our study, whereby *CRK29* was down-regulated in a Golden Gate Wax variety while up-regulated in Teebus-RR-1 variety at 14-dpi. In addition, the down-regulation of CRK genes in wheat (*Triticum aestivum*) during leaf rust (*Puccinia striiformis f.* sp. *tritici*) infection has been associated with localised cell death (Kamel et al., 2023), therefore supporting the rapid occurrence of leaf senescence in Golden Gate Wax infected with *U. appendiculatus* as observed in this study. In pathogen defense, *CRK29* has been found to play a crucial role in PAMP-triggered immunity (PTI). The up-regulation of CRKs such as *CRK29* upon pathogen perception has been found to be involved in reinforcing the cell wall and improving stomatal immunity, thus blocking pathogen haustoria development (Bourdais et al., 2015; Tang et al., 2017).

CYP450 genes have been found to participate in several metabolomic processes in plants because of their ability to reach absorption peaks of 450 nm, ideal for photosynthetic activities (Liu et al., 2022). As a result, several biochemical processes that are essential in metabolite synthesis such as terpenoids, alkaloids, lipids and others have been highlighted to be associated with CYP450 genes (Mizutani, 2012). In this study, CYP450 genes expressed in Teebus-RR-1 were all down-regulated, while only *CYP76C4* was down-regulated in Golden Gate Wax. In *Arabidopsis*, *CYP76C4* has been reported to be involved in the terpenoid (Leitão et al., 2021) and linalool metabolism (Banerjee and Hamberger, 2018). The function of *CYP76C4* and its regulation under pathogen attack in common bean still requires more studies. Interestingly, *CYP82C4* was down-regulated in Teebus-RR-1 while up-regulated in Golden Gate Wax. The down-regulation of *CYP82C4* in mutant *Arabidopsis* plants has been associated with iron deficiency and found to be positively correlated to genes that are important for early stress signaling (Murgia et al., 2011b). In this study, the up-regulation of *CYP82C4* by Golden Gate Wax at 14-dpi may indicate iron accumulation during infection, consequently contributing to feeding the *U. appendciulatus* pathogen. These findings require further studies to validate expression patterns of *CYP82C4* in susceptible varieties. Interestingly, iron withholding by the plants during pathogen infection has been found to be an immunity strategy that limits pathogen growth (Liu et al., 2021). The feeding habits of *U. appendiculatus* have not been fully studied and therefore more studies are required. Genes from the CYP94 family were also observed in our study, which have been widely reported for their contribution in mediating jasmonoyl-isoleucine (JA-Ile) catabolic pathway essential in stress signaling (Bruckhoff et al., 2016; Marquis et al., 2020). Despite their importance in JA-lle catabolic pathway, the overexpression of *CYP94B1* in maize mutant lines has been found to be involved in functions that hydrolyse JA-lle, which consequently controls flower and fruit development (Lunde et al., 2019). Based on the later observation, we hypothesize that *U. appendiculatus* infection may lead to activation of specific genes such as *CYP94B1* that may incite pathogen-accelerated early flower development as observed in Golden Gate Wax at 21-dpi.

Genes from the terpene synthase family were also observed in Golden Gate Wax and Teebus-RR-1 in response to *U. appendiculatus* infection. For example, terpene synthase 14 (*TPS14*) was down-regulated in both varieties at 14-dpi in our study. In a study evaluating the response of soybean to anthracnose (*Colletotrichum truncatum*), *TPS14* was found to be a backbone in the metabolism of diterpenes and diterpenoids (Zhu et al., 2022). The regulatory patterns of *TPS14* in response to fungal pathogen infection have not been fully described in many studies. On the other hand, *TPS21* has been found to be associated with increased levels of methyl jasmonate (MeJA), which is a crucial hormone in signaling stress such as herbivore and pathogen attack (Rodriguez-Saona et al., 2013). In this study, *TPS21* was greatly up-regulated in the Golden Gate Wax when compared with Teebus-RR-1. Although this may seem like an ideal gene expression pattern, increased expressions of MeJA has been found to result in poor plant growth (Kurowska et al., 2020), reduced seed production and accelerated defoliation (Cipollini, 2007), which could possibly explain the accelerated leaf defoliation rate observed in Golden Gate Wax in our study post evaluation at 14-dpi.

The genetic regulation of cell death in plants plays a crucial role in stress response, immunity and the multicellular development of the plant (Escamez et al., 2019). BIA genes are structurally diverse and have been found to participate in xylem differentiation and enhances wounding induced regulated cell death (Aguilera et al., 2022). For example, a decrease in the expression of in *Arabidopsis* plants exposed to mechanical wounding has been associated with enhanced plant cell death (Escamez et al., 2019). In this study, BIA was down-regulated in Golden Gate Wax at 21-dpi and from the phenotypic observation (**Figure 1B**), the variety was undergoing secondary infection possibly indicating the likelihood of cell death, post evaluation at 21-dpi.. However, the biological regulation of *BIA* in legume plants under pathogen attack still requires more exploration. Other genes that were expressed in the two varieties include the WRKY*, MBF1C and ROF2* genes, among others. WRKY has been known to be transcriptional modulators when plants are exposed to stress as they are regulators of PTI and ETI to several pathogens (Ramos et al., 2021). In this study, a down-regulation of *WRKY70* was observed in Teebus-RR-1 at 21-dpi, possibly suggesting that there was synthesis of JA genes while the opposite was observed in Golden Gate Wax. *WRKY70* is a transcription factor that promotes the production of salicylic acid (SA) and jasmonic acid (JA) signalling pathways in plant defence (Zhou et al., 2018). For example, in chickpeas (*Cicer arietinum*) infected with fusarium wilt (*Fusarium oxysporum*), an increased expression of *WRKY70* was found to be associated with plant susceptibility to the pathogen (Chakraborty et al., 2020), thus confirming the findings of this study with similar *WRKY70* expression patterns in Golden Gate Wax.

Interestingly, *MBF1C* and *ROF2* were found to be up-regulated in the Teebus-RR-1 variety, compared to the Golden Gate Wax. In potato (*Solanum tuberosum*) infected with brown rot (*Ralstonia solanacearum*), *MBF1C* was found to be incited by treatment with plant hormones SA, JA and ABA, thus improving plant defence (Yu et al., 2021). Furthermore, in *Arabidopsis*, increased expression of *MBF1C* was found to play a defence role against fungal pathogens gray mould (*Botrytis cinerea*) and bacterial canker (*Pseudomonas syringae*) (Wang et al., 2019). In this study, although there was no hormone treatment, the expression of *MBF1C* was up-regulated in Teebus-RR-1, possibly indicating defence response to the pathogen, unlike Golden Gate Wax which down-regulated the gene. In addition, an up-regulation of *ROF2* in transgenic *Arabidopsis* plants infected with *P. syringe* was found to reduce pathogen growth by up to 40% (Pogorelko et al., 2014). The regulation pattern of this gene in Teebus-RR-1 may suggest the variety's ability to prohibit *U. appendiculatus* growth. Therefore, *ROF2* can be used as a reference gene for *U. appendiculatus* resistance breeding initiatives. Teebus-RR-1 can also be used as a reference variety for resistance to *U. appendiculatus* to produce potential resistant transgenic lines using the genome editing tool CRISPR-Cas9 as also explored in tomato (Santillán Martínez et al., 2020) and soybean (Bui et al., 2023) for powdery mildew resistance.

### 4.2 Potential biomarker genes of common bean for resistance to *U. appendiculatus* race 31-1

When exposed to stress conditions, plants express numerous genes that are tuned positively and negatively to balance general plant growth and development (Roy et al., 2018). Several genes that have been reported for pathogen response in plants were identified in this study. For example, pathogen response genes *RPS2* and *CAR1* from the *NB-ARC* domain were observed in both varieties at 14- and 21-dpi. *RPS2* and *CAR1* enable recognition of pathogen invasion by using resistant or R proteins that activate ETI as a secondary line of defence. The R proteins contain LRR domain that is merged into a composite central nucleotide binding NB-ARC that restricts pathogen activity (van Ooijen et al., 2008). In this study, both *RPS2* and *CAR1* were down-regulated in both varieties. It is worth noting that at 14-dpi, *RPS2* and *CAR1* were exponentially down-regulated in Golden Gate Wax, meanwhile Teebus-RR-1 moderately down-regulated the genes. The transient expression of *RPS2* in tobacco (*Nicotiana benthamiana*) leaves in response to crown gall (*Agrobacterium tumefaciens*) has been found to cause cell death (Ngou et al., 2022). The observed expression levels of *RPS2* in this study could indicate extreme and prolonged down-regulation of the gene in Golden Gate Wax at 14-dpi, consequently resulting in cell death/hypersensitive reaction (HR). Interestingly, *LRRAC1*, has been found to be a signaling motif that contributes to plant pathogen resistance and is considered a “gate keeper” (Fang et al., 2022). In this study, the interaction between LRR and NB-ARC domains resulted in the expression of *LRRAC1*. The interaction of these two domains has been found to cause gene de-repression (Wang et al., 2015), possibly disrupting the inhibition of HR which resulted in slow cell death, elucidation leaf defoliation in Golden Gate Wax. Another interesting gene, *DM2H* which encodes disease resistant protein of the TIR-NBS-LRR class was also observed. In *Arabidopsis*, *DM2H* has been found to induce autoimmunity under disease pressure through its interaction with gene products that have incompatible alleles with the gene, therefore causing disturbance in “guardee” gene pairs (Ordon et al., 2021). In this study, Golden Gate Wax up-regulated the *DM2H* at 14-dpi, meanwhile down-regulated in Teebus-RR-1. Therefore, we hypothesize that at 14-dpi, gene expression regulation of specific genes that may be responsible for pathogen resistance should be down-regulated to avoid interference with the synthesis of other specific defence related genes. The occurrence of R genes and their regulation in legumes is complex and different in comparison to other crops (Angelo et al., 2023). Further studies detailing the gene expression patterns of genes that are crucial for pathogen resistance are important.

The expression of *CYP93D1* has been linked to the synthesis of flavonoids in the plant’s functional metabolomic processes (St-Pierre and De Luca, 2000) and in this study, *CYP93D1* was not expressed in Golden Gate Wax while down-regulated in Teebus-RR-1. In addition, the synthesis of JA in maize (Dou et al., 2021) has been found to be related to the expression of *CYP93D1*. This may suggest that molecule signaling, biological function, vascular bundle transmission and other JA processes were affected by *U. appendiculatus* in Golden Gate Wax. Similar down-regulation patterns of *CYP82C4* were observed in both varieties, and in *Arabidopsis*, *CYP82C4* has been found to be correlated with the accumulation of iron (Fe) together with the plant’s general plant circadian rhythm (Murgia et al., 2011a). In this experiment, although there was a down-regulation of *CYP82C4* in both varieties at 14-dpi in response to *U. appendiculatus*, in Teebus-RR-1 the gene was moderately down-regulated in comparison Golden Gate Wax which highly down-regulated. It has been suggested that prioritising *P. vulgaris* varieties that express high iron concentrations is beneficial, as these varieties tend to demonstrate an increased ability to withstand pathogens (Islam et al., 2002).

Pathogen infection results in plant DNA damage that is mediated by the expression of *ABO4*, a gene that belongs to the DNA polymerase epsilon catalytic subunit responsible for DNA replication, cell cycle control and flower development (Wickramasuriya et al., 2022). In this study, both varieties down-regulated *ABO4* at 14-dpi. However, at 21-dpi, the gene was extensively up-regulated by 20-fold in Golden Gate Wax, while up-regulated by only 2-fold in Teebus-RR-1. A similar expression pattern of terpene gene *ATTPS-CIN* was observed across varieties at both time points. The role of these genes in plant-pathogen interactions is not widely reported, hence a need for further investigations.

### 4.3 Fungal categories, pathway activation and gene localisation in response to *U. appendiculatus*

Metabolomic pathways were highly enriched by Golden Gate Wax at 14-dpi by >150 genes. Metabolic pathway includes several biochemical reactions that are responsible for signal transduction, primary and secondary metabolism among others (Xiong et al., 2023). In wheat varieties infected with rust caused by *Puccinia graminis* f. sp. *tritici* (*Pgt*), the occurrence of metabolic pathways was found to be associated with enhancement of pathogen virulence, development and secretion (Kataria and Kaundal, 2022).We hypothesise that Golden Gate Wax possibly lacked targeted defence mechanisms and only enriched general biological activities such as metabolic pathways, and biosynthesis of secondary metabolites. Pathways expressed by Teebus-RR-1 at 14-dpi, particularly protein processing in the endoplasmic reticulum, sequence binding DNA, response to stress, hormone mediated signaling and others indicate that *U. appendiculatus* infected varieties deploy a series of multiple defence-related pathways to regulate pathogen stress. In watermelon (*Citrullus lanatus*), enrichment of response to stress pathway was found to contribute to reducing toxin synthesis of *F. oxysporum f.* sp (Wang et al., 2022). In addition, the enrichment of hormone signaling pathways has been found to play a role in forming hormone networks that assist in mediating stress and promote plant growth (Verma et al., 2016). Pathways enriched in Teebus-RR-1 at 14-dpi indicate that the variety expressed specific genes that were geared at enriching pathogen response pathways. Xyloglucan:xyloglucosyl transferase activity was among the two enriched pathways in Golden Gate Wax at 21-dpi. Enrichment of xyloglucan:xyloglucosyl transferase activity has been found to be associated with cell wall re-modelling, cell wall reinforcement, signaling, and apoplastic barrier formation (Wang et al., 2021). This may suggest that at 21-dpi, Golden Gate Wax was re-enforcing its cell wall even with eminent re-infection of secondary leaves. In Teebus-RR-1 at 21-dpi there was increased photosynthetic activity from pathways including thylakoid, photynth thylakoid, photosynthetic membrane and chloroplast. The enrichment of photosynthetic pathways such as the latter has been found to play a role in chloroplast enrichment while degradation is observed in susceptible varieties (Kumar and Kirti, 2015). Findings of enrichment trends observed in Golden Gate Wax and Teebus-RR1 are like the elucidations that have been previously made.

In this study, DEGs and biomarkers were found to be associated with rust resistance, and these were localised on chromosomes *Pv04* (*MBF1C*: *Phvul.004G162100*), *Pv06* (*RPS2*: *Phvul.006G038100* and *ABO4*: *Phvul.006G055950*), *Pv09* (*CYP82C4*: *Phvul.009G061450*) and *Pv11* (*LRRAC1*: *Phvul.011G197800*, *ATTPS-CIN*: *Phvul.011G107933* and *TPS21*: *Phvul.011G143100*), among others. In comparison to reported resistant genes, there were no unigene matches and thus no previously reported Ur genes were linked to resistance in both varieties. Previously, resistance has been majorly found to be linked to *Pv11* and *Pv04* among others (Arunga et al., 2012; Devi et al., 2024). In this study, the potential biomarkers identified were localised on *Pv11* and *Pv04*, thus consistent with the previous studies. However, previous findings have only linked resistance to specific genes found at a specific chromosome. The use of RNA-Seq in this study evidently showed that pathogen resistance is linked to several genes localised in different chromosomes, expressed differentially and timely as a response strategy to *U. appendiculatus*.

## 5 Conclusion

In this study we present the integration of morphology and gene expression patterns of common bean varieties with varying resistance response when exposed to rust. Defense mechanisms in varieties are associated with timely response to pathogen attack. At 14-dpi, rust was highly virulent and Golden Gate Wax expressed an extensive number of genes that were not geared at aiding the plant’s defence mechanism. For plants to achieve immunity to pathogens, timely cell-cell communication is crucial as this consequently impacts coordination of pathways. The evaluation of varieties at crucial pathogen infection stages can aid greatly in identifying pathogen stress signaling pathways that can be used as indicators of resistance for subsequent breeding and crop improvement. Late-stage evaluations of pathogen resistance are critical for assessing the ability of plants to sustain defence under real-world farming conditions, as seen with common bean rust, which becomes evident only at later growth stages. Aligning genomic resistance screening with practical, field-relevant timelines instead of laboratory focused early timepoints ensures that breeding efforts deliver durable and farmer-usable crop varieties. In addition, the integration of biomarker discovery in transcriptome data analysis pipelines can be beneficial in selecting pathogen resistant genes that can further be introduced to other lines. The gene expression assemblage patterns observed in this study used by common bean varieties in response to rust can be used for genetic detection of long-term resistance. Intensifying research studies that use techniques such as transcriptomics to uncover defence mechanisms in legumes while integrating other NGS technologies can aid in accelerating breeding efforts aimed at pathogen resistance. For example, gene knockout and amplification techniques can be used to improve variety resistance. Furthermore, evaluation of other *P. vulgaris* varieties in response to more or a cocktail of strains of *U. appendiculatus* is planned in the future to gain more insights on plant-pathogen interactions. Assembly and publication of the *U. appendiculatus* genome will also aid in elucidating the genetic mode of attack of the pathogen while also providing a genomic comparison basis for both the plant and pathogen.

## Data Availability

The datasets presented in this study can be found in online repositories. The names of the repository/repositories and accession number(s) can be found below: https://www.ncbi.nlm.nih.gov/, PRJNA1061833.

## Supplementary material

Supplementary Figure 1 Phenotypic preliminary evaluation of other varieties in response to *U. appendiculatus* race 31-1 at early vegetative stage (14-dpi). GN1140 **(A)**, Early Gellatin **(B)**, Golden Gate Wax **(C)**, PI181996 **(D)**, Teebus-RR-1 **(E)** and Mexico 309 **(F)** at 14-dpi.

Supplementary Figure 2 OPLS representation of gene expression data across all treatments. Denotations on figure are G: Golden Gate Wax, T: Teebus-RR-1, 14dpi: 14 days post infection, 21dpi: 21 days post infection, digits after variety symbols, 0: control/mock inoculated, and 1: race 31-1.

Supplementary Figure 3 Heatmap showing DEG’s across all sample groups. The upper colour denoting the sample classes of Golden Gate Wax (G), Teebus-RR-1 (T), race 31-1 denotated as 1 and control denoted as 0 after the variety denotation at the two time points 14- and 21-dpi.

Supplementary Figure 4 Logarithmic relative expression (y-axis) of genes from varieties Golden Gate Wax and Teebus-RR-1 at both time points (14- and 21-dpi) from the RNA-Seq and qRT-PCR analysis. Common DEGs across varieties and treatments **(A)** and Biomarkers expressed across varieties and treatments **(B)**.

Supplementary Table 1 Unigene names of Ur genes previously characterised in Meso-American (MA) and Andean (A) varieties with chromosome locations of *U. appendiculatus* the mapped resistant genes using marker technologies.

Supplementary Table 2 Number of raw sequence reads and % GC content of all analysed treatment samples of the experiment.

Supplementary Table 3 Regulation patterns of DEGs in varieties, Golden Gate Wax and Teebus-RR-1 at time points of evaluation (14- and 21-dpi).

Supplementary Table 4 Regulation patterns of DEGs that were common in Golden Gate Wax at 14- and 21-dpi.

Supplementary Table 5 Top right and bottom left quadrant potential variety biomarkers for *U. appendiculatus* race 31-1 response, obtained from the S-plot generated from SIMCA’s OPLS analysis. from the
