# Supplementary figures and images for: Insights of Phaseolus vulgaris’ response to infection by Uromyces appendiculatus using an RNA-seq approach
Source: Front Plant Sci. 2025 Jun 2;16:1557954. doi: 10.3389/fpls.2025.1557954 (PMC12171439; doi:10.3389/fpls.2025.1557954)

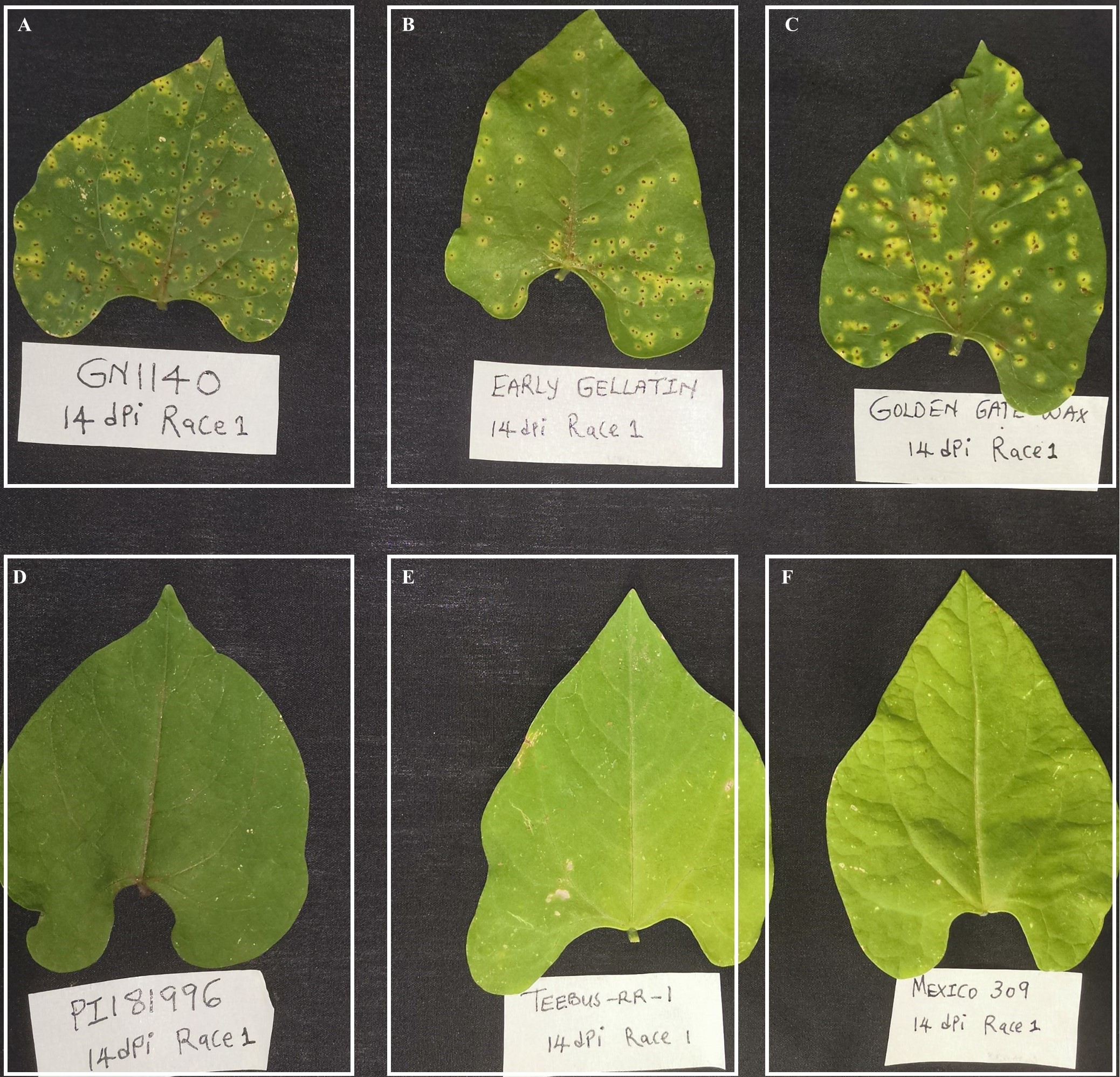

Supplement: Supplementary Figure 1 — Phenotypic preliminary evaluation of other varieties in response to U. appendiculatus race 31-1 at early vegetative stage (14-dpi). GN1140 (A), Early Gellatin (B), Golden Gate Wax (C), PI181996 (D), Teebus-RR-1 (E) and Mexico 309 (F) at 14-dpi. [file Image1.jpeg]

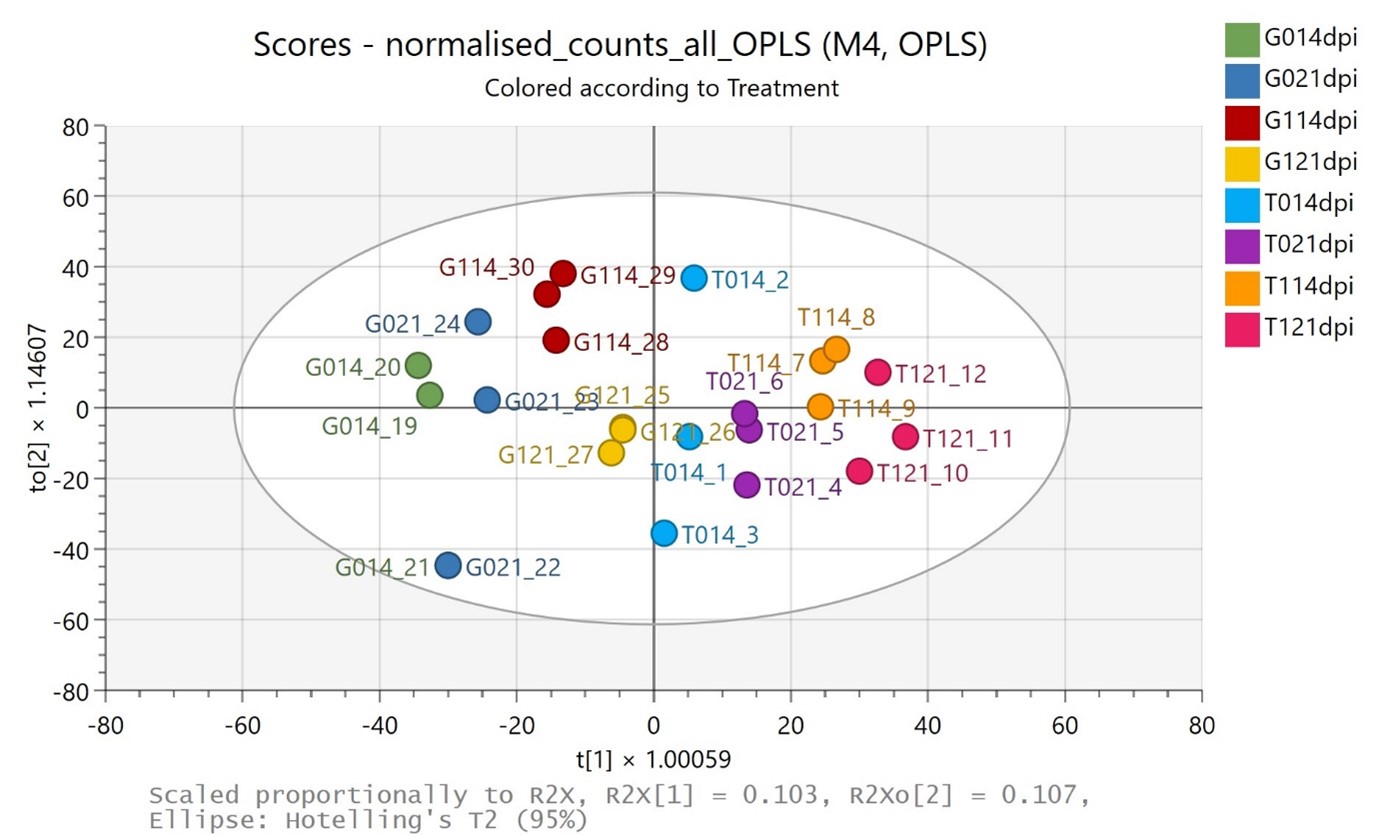

Supplement: Supplementary Figure 2 — OPLS representation of gene expression data across all treatments. Denotations on figure are G: Golden Gate Wax, T: Teebus-RR-1, 14dpi: 14 days post infection, 21dpi: 21 days post infection, digits after variety symbols, 0: control/mock inoculated, and 1: race 31-1. [file Image2.jpeg]

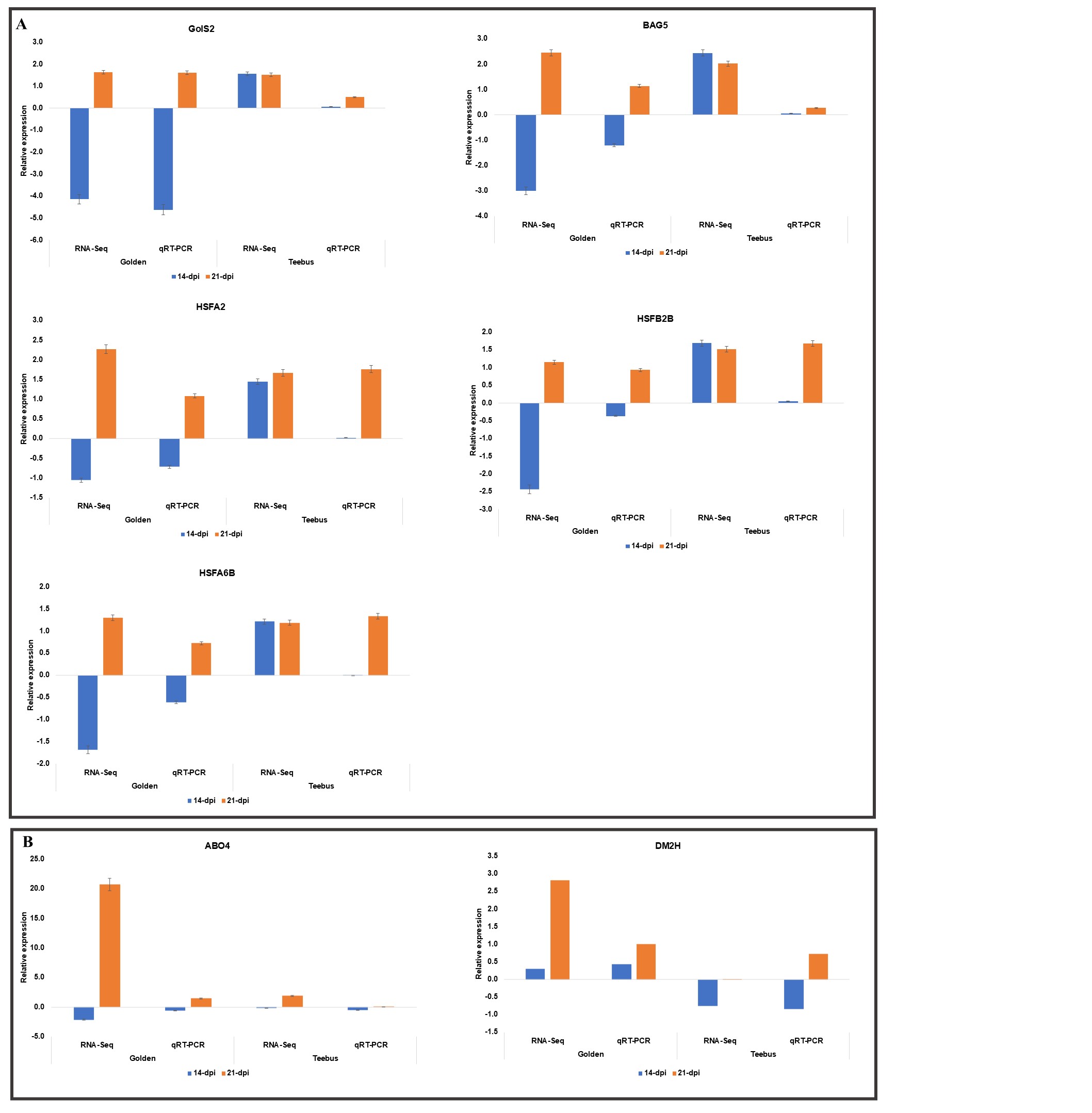

Supplement: Supplementary Figure 3 — Heatmap showing DEG’s across all sample groups. The upper colour denoting the sample classes of Golden Gate Wax (G), Teebus-RR-1 (T), race 31-1 denotated as 1 and control denoted as 0 after the variety denotation at the two time points 14- and 21-dpi. [file Image3.jpeg]

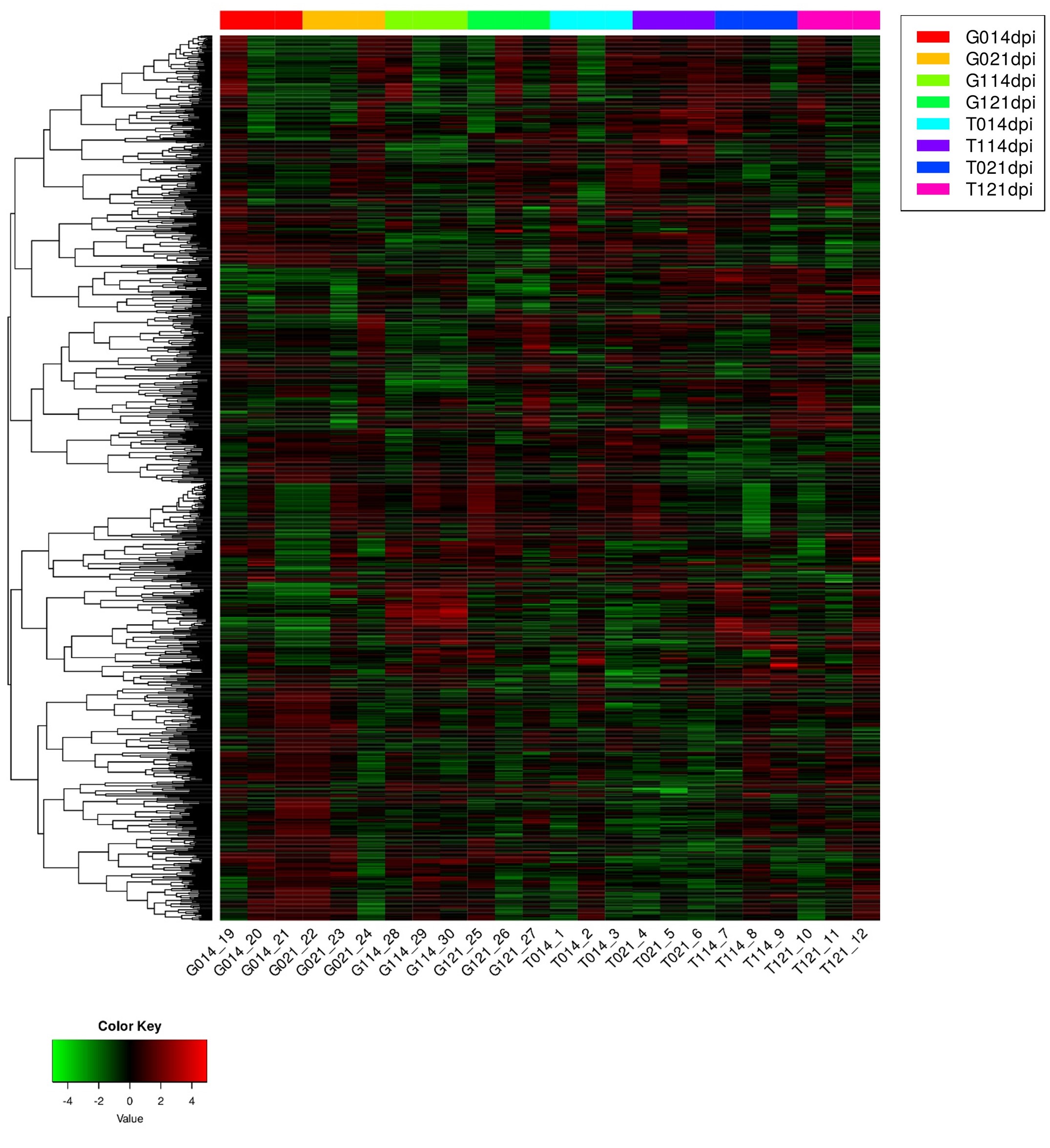

Supplement: Supplementary Figure 4 — Logarithmic relative expression (y-axis) of genes from varieties Golden Gate Wax and Teebus-RR-1 at both time points (14- and 21-dpi) from the RNA-Seq and qRT-PCR analysis. Common DEGs across varieties and treatments (A) and Biomarkers expressed across varieties and treatments (B). [file Image4.jpeg]
